# Leptin Elicits LTC_4_ Synthesis by Eosinophils Mediated by Sequential Two-Step Autocrine Activation of CCR3 and PGD_2_ Receptors

**DOI:** 10.3389/fimmu.2018.02139

**Published:** 2018-09-20

**Authors:** Natália R. T. Amorim, Tatiana Luna-Gomes, Marcos Gama-Almeida, Glaucia Souza-Almeida, Claudio Canetti, Bruno L. Diaz, Peter F. Weller, Patricia Torres Bozza, Clarissa M. Maya-Monteiro, Christianne Bandeira-Melo

**Affiliations:** ^1^Laboratório de Inflamação, Instituto de Biofísica Carlos Chagas Filho, Universidade Federal do Rio de Janeiro, Rio de Janeiro, Brazil; ^2^Departamento de Ciências da Natureza, Instituto de Aplicação Fernando Rodrigues da Silveira, Universidade do Estado do Rio de Janeiro, Rio de Janeiro, Brazil; ^3^Laboratório de Imunofarmacologia, Instituto Oswaldo Cruz-IOC, FIOCRUZ, Rio de Janeiro, Brazil; ^4^Department of Medicine, Harvard Medical School, Beth Israel Deaconess Medical Center, Boston, MA, United States

**Keywords:** leptin, eosinophil, prostaglandin D_2_, leukotriene C_4_, CCR3, CCL5, lipid body, lipid droplet

## Abstract

Leptin is a cytokine, produced mainly by mature adipocytes, that regulates the central nervous system, mainly to suppress appetite and stimulate energy expenditure. Leptin also regulates the immune response by controlling activation of immunomodulatory cells, including eosinophils. While emerging as immune regulatory cells with roles in adipose tissue homeostasis, eosinophils have a well-established ability to synthesize pro-inflammatory molecules such as lipid mediators, a key event in several inflammatory pathologies. Here, we investigated the impact and mechanisms involved in leptin-driven activation of eicosanoid-synthesizing machinery within eosinophils. Direct *in vitro* activation of human or mouse eosinophils with leptin elicited synthesis of lipoxygenase as well as cyclooxygenase products. Displaying selectivity, leptin triggered synthesis of LTC_4_ and PGD_2_, but not PGE_2_, in parallel to dose-dependent induction of lipid body/lipid droplets biogenesis. While dependent on PI3K activation, leptin-driven eosinophil activation was also sensitive to pertussis toxin, indicating the involvement of G-protein coupled receptors on leptin effects. Leptin-induced lipid body-driven LTC_4_ synthesis appeared to be mediated through autocrine activation of G-coupled CCR3 receptors by eosinophil-derived CCL5, inasmuch as leptin was able to trigger rapid CCL5 secretion, and neutralizing anti-RANTES or anti-CCR3 antibodies blocked lipid body assembly and LTC_4_ synthesis induced by leptin. Remarkably, autocrine activation of PGD_2_ G-coupled receptors DP1 and DP2 also contributes to leptin-elicited lipid body-driven LTC_4_ synthesis by eosinophils in a PGD_2_-dependent fashion. Blockade of leptin-induced PGD_2_ autocrine/paracrine activity by a specific synthesis inhibitor or DP1 and DP2 receptor antagonists, inhibited both lipid body biogenesis and LTC_4_ synthesis induced by leptin stimulation within eosinophils. In addition, CCL5-driven CCR3 activation appears to precede PGD_2_ receptor activation within eosinophils, since neutralizing anti-CCL5 or anti-CCR3 antibodies inhibited leptin-induced PGD_2_ secretion, while it failed to alter PGD_2_-induced LTC_4_ synthesis. Altogether, sequential activation of CCR3 and then PGD_2_ receptors by autocrine ligands in response to leptin stimulation of eosinophils culminates with eosinophil activation, characterized here by assembly of lipidic cytoplasmic platforms synthesis and secretion of the pleiotropic lipid mediators, PGD_2_, and LTC_4_.

## Introduction

Classically, eosinophils are perceived as innate leukocytes with important roles in allergic conditions and parasitic infection. More recently, the understanding of eosinophil biological significance has evolved from disease-driven inflammatory effector cells to immunomodulatory housekeepers (1–3). Eosinophils are now recognized to be resident cells in different tissues where they play homeostatic roles, including uterine priming for pregnancy (4) or supporting mammary gland development (5, 6). In healthy white adipose tissue, sentinel eosinophils preserve adipose homeostatic baseline and metabolic regulation, and mitigate obesity. Specifically, adipose tissue-associated eosinophils mediate M2 polarization of adipose macrophages in a paracrine fashion by releasing cytokines, such as IL-4 (7). M2 macrophage-enriched adipose tissue is a tolerogenic environment, which favors browning and limits adipose expansion (7–9). In eosinophil-deficient mice, loss of adipose tissue eosinophil population allows phenotypic switch of macrophages from M2 to M1, setting up an inflammatory environment that culminates with weight gain and systemic insulin resistance (7). Reduction of eosinophil numbers in obese adipose tissue reinforces the notion that eosinophil population is actively regulated across different physiological states of the adipose tissue (10, 11).

Amongst physiological mediators promoting eosinophil localization within adipose tissue, locally IL-5 released by innate lymphoid type 2 cells (ILC2s) (12), as well as extracellular matrix molecules (10), emerged as chief regulators of adipose tissue eosinophilia development and survival. Besides interacting with adipose resident immune cells, like ILC2 and M2 macrophages, eosinophils also adjoin lean adipocytes within healthy adipose tissue. Therefore, eosinophil/adipocyte cross-talk may also take place to maintain homeostasis and regulation of adipocyte lipid handling and storage. In agreement, adipose eosinophils were identified as cellular sources of cathecholamines, which activate adipocyte-expressed β3 adrenoceptors triggering release of adiponectin, a key regulator of adipose vascular functionality (13). However, even though it is becoming clear that eosinophil activation represents a lead-off event of steady state adipose tissue maintenance, very little is known about the local molecular signals that control cellular activity of eosinophils within lean adipose tissue.

Adipokines are constitutively secreted by adipocytes, with both hormonal and *in situ* functions. They may significantly modulate adipose eosinophil roles since eosinophils express specific adipokine receptors, like adiponectin AdipoRs (14) and leptin ObRs receptors (15). Like other leukocytes, eosinophils express the active isoform of leptin receptors ObRb (15–17), which typically signals via PI3K-activated pathways (18–20). Acting in a variety of tissues, adipocyte-derived leptin has pleiotropic effects, notably the regulation of lipid metabolism. In eosinophils, ObRb activation by leptin is known to increase cell survival, chemokinesis and secretion of pro-inflammatory cytokines (15–17). Of note, eosinophils have diverse immune functional capabilities, not restricted to cytokine secretion. Eosinophils are particularly capable of producing bioactive lipids from arachidonic acid metabolism within their cytoplasmic lipid bodies, including prostaglandin (PG)E_2_ and PGD_2_ and leukotriene (LT)C_4_ (21, 22). Acting on specific receptors with widespread tissue expression (including adipose tissue; (23), these lipid mediators can mediate functions, from homeostatic to pro-inflammatory, as diverse as eosinophils themselves. Pertinent here, leptin prompts 5-lipoxygenase-mediated synthesis of LTB_4_ within newly formed cytoplasmic lipid bodies in macrophages (24). Studies of eosinophil activation by adipocyte-derived factors, like leptin, are germane for full characterization of the potential mechanisms involved in eosinophil-driven contribution to adipose tissue homeostasis. Here, we investigated leptin's ability to elicit arachidonic acid metabolism within eosinophils, evaluating the cellular signaling involved. Specifically, by studying the mechanisms of leptin-induced LTC_4_ synthesis in both human and mouse eosinophils, we uncovered a leptin-triggered complex signaling pathway, which comprises two consecutive and rapid autocrine loops within eosinophils, including up-stream CCL5 release/CCR3 activation followed by PGD_2_ release/DP receptor activation.

## Materials and methods

### Isolation of human blood eosinophils

Peripheral blood was obtained with informed consent from normal donors. Briefly, after dextran sedimentation and Ficoll gradient steps, eosinophils were isolated from contaminating neutrophils by negative immunomagnetic selection using the EasySep™ system (StemCell Technologies Inc.) (cell purity ~99%; cell viability ~95%). The protocol was approved by ethical review boards of both the Federal University of Rio de Janeiro and the Oswaldo Cruz Foundation (Rio de Janeiro, Brazil).

### *In vitro* eosinophil differentiation from mouse bone marrow cells

BALB/c mice from both sexes were used. Animals were obtained from the Oswaldo Cruz Foundation breeding unit (Rio de Janeiro, Brazil). The protocols were approved by both Federal University of Rio de Janeiro Animal Use and Oswaldo Cruz Foundation Animal Welfare Committees. Eosinophils were differentiated *in vitro* from mouse bone marrow cells as previously described (25). Briefly, bone marrow cells were collected from femurs and tibiae of wild-type BALB/c mice with RPMI 1640 containing 20% FBS. Cells were cultured at 10^6^ cells/mL in RPMI 1640 containing 20% FBS (VitroCell), 100 U/mL penicillin, 10 μg/ml streptomycin, 2 mM glutamine and 1 mM sodium pyruvate (Sigma), 100 ng/mL stem cell factor (SCF; PeproTech) and 100 ng/mL FLT3 ligand (PeproTech) from days 0 to 4. On day 4, SCF and FLT3-L were replaced with IL-5 (10 ng/mL; Peprotech). On day 14, eosinophils were enumerated (purity ≥ 90%).

### *In vitro* eosinophil stimulation and treatments

Purified human eosinophils or mouse eosinophils at 2 × 10^6^ cells/mL or 3 × 10^6^ cells/mL in Ca^2+^/Mg^2+^ HBSS (HBSS^+/+^; pH 7.4) were pre-treated with the PI3K inhibitors wortmannin (1 μM; Biomol) and LY294002 (10 μM; Cayman Chemicals), PKC inhibitor calphostin C (1 μM; Biomol), pertussis toxin (PTX; 100 ng/mL), neutralizing monoclonal antibodies anti-CCL5 (10 μg/mL) and anti-CCR3 (10 μg/mL) (both from R&D), the PAF receptor antagonist BN52021 (10 μM), PGD_2_ receptor antagonists BWA868c (200 nM; DP1 receptor) and Cay10471 (200 nM; DP2 receptor, or PGD_2_ synthesis inhibitors HQL-79 (10 μM; H-PGDS) and AT-56 (10 μM; L-PGDS) (all from Cayman Chemicals) at 37°C for 30 min before stimulation with human recombinant (hr) or mouse recombinant (mr) leptin (0.5, 5, or 50 nM, as indicated; Peprotech) for 15 or 60 min in a water bath (37°C). Alternatively, eosinophils were also stimulated with PAF (1-*O*-hexadecyl-2-acetyl-*sn*-glyceryl-3-phosphorylcholine; 1 μM; Cayman Chemicals), hr CCL5 (also known as RANTES−100 ng/mL; R&D) or PGD_2_ (25 nM; Cayman Chemicals). Each experimental condition was repeated at least three times with eosinophils purified from different donors or differentiated *in vitro* from different mouse bone marrows.

### EicosaCell for intracellular immunodetection of LTC_4_

EicosaCell methodology (26, 27) was used to immunolocalize intracellular LTC_4_ at its synthesis sites. *In vitro*-stimulated human eosinophils were mixed with an equal volume of water-soluble 1-ethyl-3-(3-dimethylamino-propyl) carbodiimide (EDAC; 0.5% in HBSS for 10 min) (Sigma), used to cross-link eicosanoid carboxyl groups to amines in adjacent proteins. After, eosinophils were cytospun onto glass slides and after block step, they were incubated with rabbit anti-LTC_4_ Abs (Cayman Chemicals) overnight and secondary DyLight488 green fluorochrome anti-rabbit IgG (Jackson ImmunoResearch Laboratories) for 1 h. As specificity controls for the immunolocalization of LTC_4_, rabbit IgG (Sigma) was routinely included as a nonimmune control for the primary anti-LTC_4_ and eosinophils were pre-treated with PI3K inhibitor LY294002 (10 μM), for 30 min prior leptin stimulation. Mounting medium containing DAPI was applied to each slide before coverslip attachment to allow visualization of blue-stained eosinophil nuclei. Images were obtained using an Olympus BX51 fluorescence microscope at 100x magnification and photographs were taken with the Olympus 72 digital camera (Olympus Optical Co., Tokyo, Japan) in conjunction with CellF Imaging Software for Life Science Microscopy (Olympus Life Science Europa GMBH, Hamburg, Germany).

### Lipid body staining and enumeration

For lipid body enumeration within eosinophil cytoplasm, cytospun cells were fixed in 3.7% formaldehyde (diluted in HBSS^−/−^), rinsed in 0.1 M cacodylate buffer (pH 7.4), stained with 1.5% OsO_4_ for 30 min, rinsed in distilled H_2_O, immersed in 1.0% thiocarbohydrazide for 5 min, rinsed in 0.1 M cacodylate buffer, restained with 1.5%OsO_4_ for 3 min, rinsed in distilled water, and mounted. Lipid bodies were enumerated by light (osmium staining) microscopy. Fifty consecutively scanned eosinophils were evaluated in a blinded fashion by more than one individual, and the results were expressed as the number of lipid bodies *per* eosinophil. Alternatively, cells were stained with Oil Red O (lipid fluorescent stain) for better visualization of the cytoplasmic distribution of eosinophil lipid bodies. Briefly, cytospun cells were fixed in 3.7% formaldehyde (diluted in HBSS^−/−^), rinsed with 60% isopropanol, stained with 0.3% Oil Red O and rinsed with 60% isopropanol. Finally, cytospun cells were washed with water and mounted with DAPI for visualization of blue-stained eosinophil nuclei.

### Eicosanoids and CCL5 quantification

PGE_2_, PGD_2_, or LTC_4_ found in eosinophil supernatants were measured by commercial EIA kits, according to the manufacturer's instructions (Cayman), while mouse and human CCL5 were measured by commercial ELISA kits, according to the manufacturer's instructions (Petrotech). Of note, we found about 150 ng of preformed stores of CCL5 within non-stimulated *in vitro* differentiated mouse eosinophils analyzed in whole cells lysates of 3 × 10^6^ pelleted mouse eosinophils by ELISA.

### Statistical analysis

Results are expressed as the mean ± SEM and were analyzed statistically by means of ANOVA, followed by Student-Newman-Keuls test, with the level of significance set at *p* < 0.05.

## Results

### Leptin activates LTC_4_-synthesizing machinery within eosinophils *in vitro*

**Figure 1:**
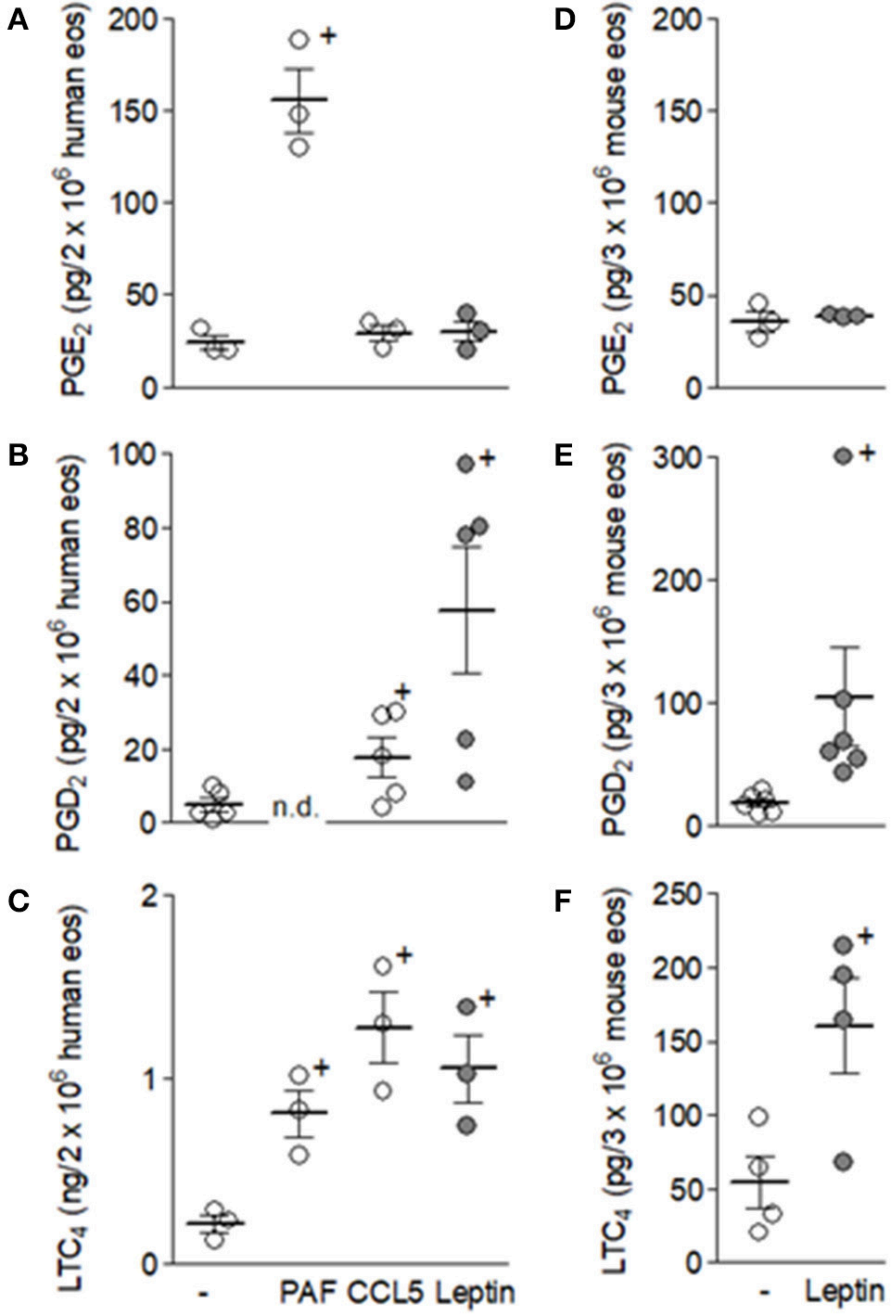
Leptin triggers PGD_2_ and LTC_4_ synthesis, but not PGE_2_, within eosinophils. Human eosinophils **(A–C)** were stimulated with PAF (1 μM), hrCCL5 (100 ng/mL), or hrleptin (50 nM) for 1 h. Mouse eosinophils **(B–F)** were stimulated with mrleptin (50 nM) for 1 h. Eicosanoid PGE_2_, PGD_2_, or LTC_4_ production by eosinophils were quantified in cell supernatants by specific EIA kits. Values are expressed as the mean ± SEM of at least three distinct donors or three different mouse bone marrow cultures. + *p* < 0.05 compared with non-stimulated eosinophils.

Human eosinophils upon stimulation preferentially synthesize LTC_4_ as an arachidonic acid metabolite by activating the 5-LO pathway, although it is well established that proper intracellular signaling within eosinophils can also couple to COX-driven prostanoid synthesis (22). Here, we initially examined whether leptin can directly activate LTC_4_ synthesis within human as well as mouse eosinophils *in vitro*, also evaluating its potential effect on prostanoid synthesis. As shown in Figure 1, while leptin (50 nM) failed to trigger PGE_2_ synthesis within both human and mouse eosinophils (Figures 1A,D), the adipokine induced rapid (within 1 h) PGD_2_ (Figures 1B,E) and LTC_4_ synthesis (Figures 1C,F) by both eosinophil species studied, indicating that leptin-elicited intracellular signaling in eosinophils activates both leukotriene- and prostanoid-synthesizing pathways. However, under leptin-stimulation eosinophil COX preferentially couples to H-PGDS rather than E series-synthesizing isomerases. Of note, eosinophils are capable of PGE_2_ synthesis upon proper stimulation, such as with PAF–an eosinophil stimulus that triggers a different profile of eicosanoid synthesis by eosinophils with production of PGE_2_ (Figure 1A) and LTC_4_ (Figure 1C), but not PGD_2_ (Figure 1B) (28, 29). On the other hand, Figure 1 also shows that CCL5 stimulation display a panel of synthesized eicosanoids (including secretion of PGD_2_ and LTC_4_, but not PGE_2_) similar to that triggered by leptin in human or mouse eosinophils. Altogether, the data illustrate the complexity and stimulus-dependent specificity of receptor-initiated arachidonic acid metabolic pathways within eosinophils, while also demonstrating that human and mouse eosinophils, at least to what concerns the ability to synthesize eicosanoids, present the same general patterns of response.

**Figure 2:**
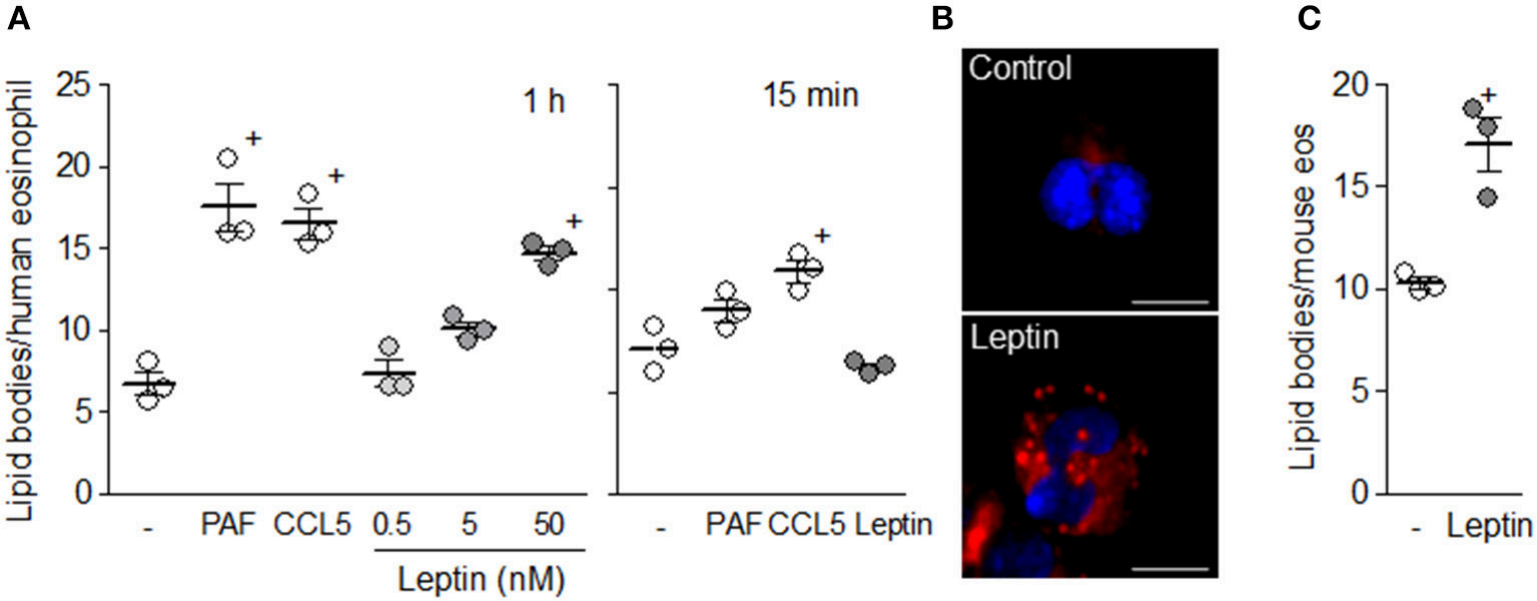
Leptin triggers lipid body biogenesis within eosinophils. In **(A)** human eosinophils were stimulated with PAF (1 μM), hr CCL5 (100 ng/mL), or hr leptin (0.5–50 nM) for 1 h or 15 min, as indicated. **(B)** shows lipid bodies stained by Oil Red O within eosinophils stimulated with hr leptin (50 nM) for 1 h. In **(C)** mouse eosinophils were stimulated with mr leptin (50 nM) for 1 h. In **(A)** and **(C)** lipid bodies were enumerated in 50 consecutive osmium-stained cells. Values are expressed as the mean ± SEM of at least three distinct donors or three different mouse bone marrow cultures. + *p* < 0.05 compared with non-stimulated eosinophils.

In parallel with increased PGD_2_ and LTC_4_ synthesis within 1 h of stimulation, leptin (50 nM) was able to directly increase the number of cytoplasmic lipid bodies within human eosinophils (Figure 2A). Morphology and distribution analysis of newly assembled lipid bodies within leptin-stimulated eosinophils visualized in either osmium (not shown) or Oil Red O stained cells (Figure 2B) revealed discrete punctate organelles with cytoplasmic localizations both adjacent to and far from nuclei. Such leptin-induced rapid lipid body biogenesis was dose-dependent and displayed levels similar to those induced by well-established eosinophil-relevant stimuli, such as CCL5 and PAF within human eosinophils (Figure 2A). Figure 2C shows that, similar to human eosinophils, mouse eosinophils also respond directly to leptin stimulation with increased assembly of these organelles—cytoplasmic platforms responsible for compartmentalization of bioactive enzymatic machinery of eicosanoid synthesis under inflammatory stimulations and markers of leukocyte activation (22). Of note, we have previously shown that *in vitro* differentiated mouse eosinophils also respond to PAF or CCR3 activation with rapid (1 h) assembly of lipid bodies, which function as intracellular compartments of eicosanoid synthesis (29).

### Leptin-induced activation of lipid body-driven LTC_4_-synthesizing machinery within eosinophils is a PI3K-dependent phenomenon

**Figure 3:**
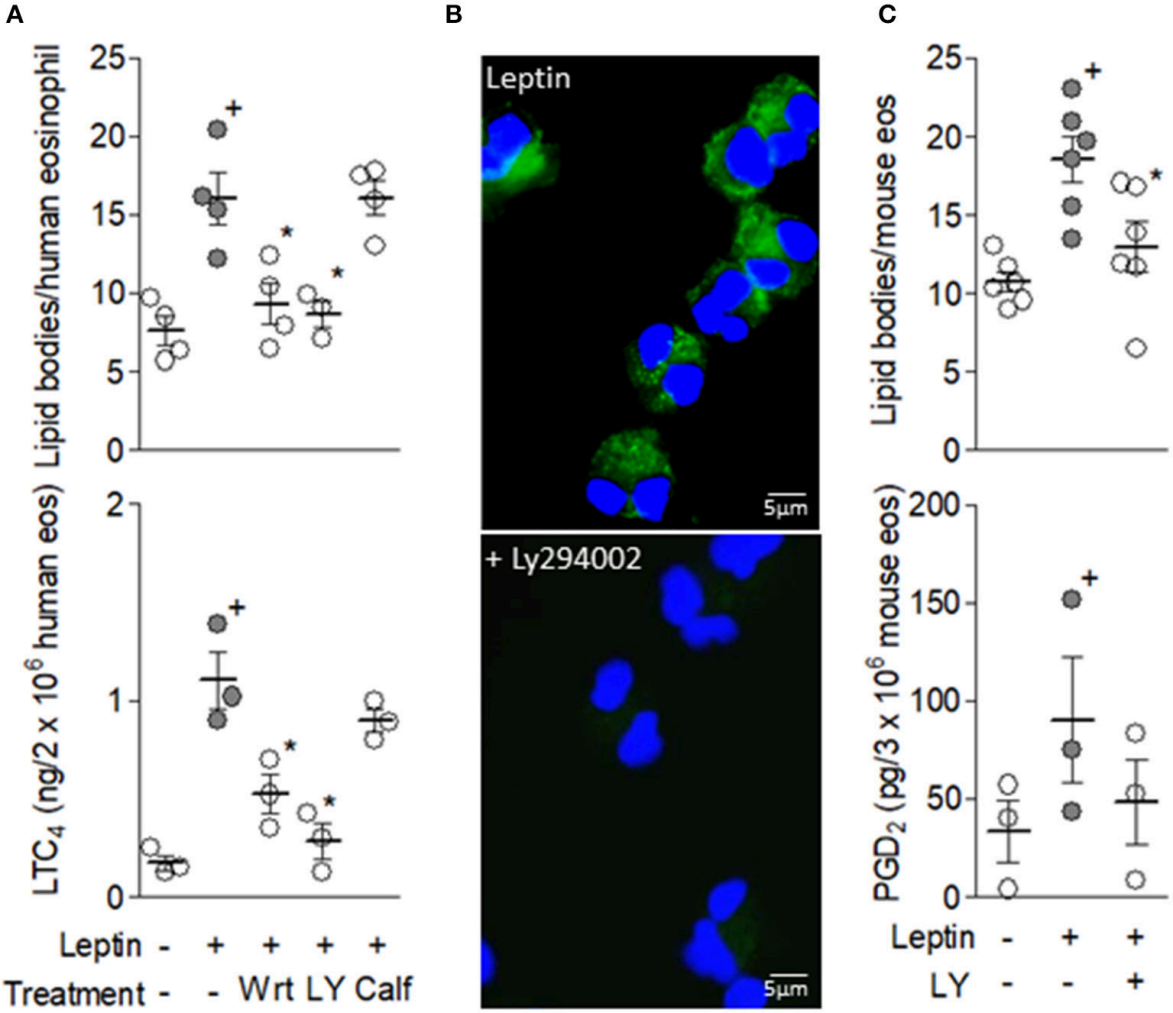
Leptin-induced lipid body-driven LTC4 synthesis depends on PI3K activation. In **(A)** human eosinophils were pretreated with PI3K inhibitors (1 μM wortmannin or 10 μM LY294002) or a PKC inhibitor (calphostin C; 1 mM) 30 min before stimulation with hr leptin (50 nM) for 1 h. **(B)** shows confocal images overlays of intracellular EicosaCell immuno-detection of newly formed LTC_4_ (green) and DAPI stained nuclei (blue) within hr leptin-stimulated (upper image) or LY294002-treated hr leptin-stimulated (bottom image) human eosinophils. In **(C)** mouse eosinophils were pretreated with PI3K inhibitor LY294002 (10 μM) for 30 min before stimulation with mr leptin (50 nM) for 1 h. Lipid body count was evaluated in osmium-stained cells and LTC_4_ production in cell-free supernatants by EIA kits. Values are expressed as the mean ± SEM of at least three distinct donors or three different mouse bone marrow cultures. + *p* < 0.05 compared with control. ^*^p < 0.05 compared with leptin-stimulated eosinophils.

Inasmuch as activation of PI3K represents an ubiquitous event of ObR-elicited intracellular signaling pathways mediating activation of different leukocyte functions (20), we evaluated PI3K involvement in leptin-induced lipid body-driven LTC4 synthesis by eosinophils. As shown in Figure 3A, leptin-stimulated human eosinophils pre-treated with two structurally non-related PI3K inhibitors (30), wortmannin or LY294002, exhibited decreased lipid body biogenesis and LTC4 production. Of note, inhibition of PKC activation by calphostin C showed no effect on leptin-induced lipid body-driven LTC4 synthesis within eosinophils (Figure 3A), even though leptin effects on macrophages depend on PKC (31). To further study the role of PI3K in LTC4 production triggered by leptin within human eosinophils, we employed the EicosaCell methodology—an imaging—and immunofluorescent-based assay that identifies spatiotemporal intracellular synthesis of lipid mediators (27). Inhibition of PI3K by LY294002 blocked immunolocalization of leptin-induced newly synthesized LTC4 (detected by a green fluorochrome labeled anti-LTC4 specific antibody) within punctate cytoplasmic compartments, which are compatible with eosinophil lipid bodies in size, form and intracellular distribution (Figure 3B), therefore establishing that lipid body-compartmentalized LTC4 synthesis is the target of PI3K inhibition. Moreover, LY294002 was also able to inhibit leptin-induced cytoplasmic lipid body biogenesis and PGD2 synthesis (Figure 3C) within mouse eosinophils, indicating that leptin-elicited lipid body-driven eicosanoid synthesis is an ObR-driven PI3K-dependent phenomenon that is conserved in both human and mouse eosinophils. Comparison between eosinophils from both species is relevant since at times, mouse and human eosinophils demonstrate differing functional responses to the same stimulatory condition (32).

### Activation of G protein-coupled CCR3 receptor by eosinophil-derived RANTES mediates leptin-induced lipid body-driven LTC_4_ synthesis

**Figure 4:**
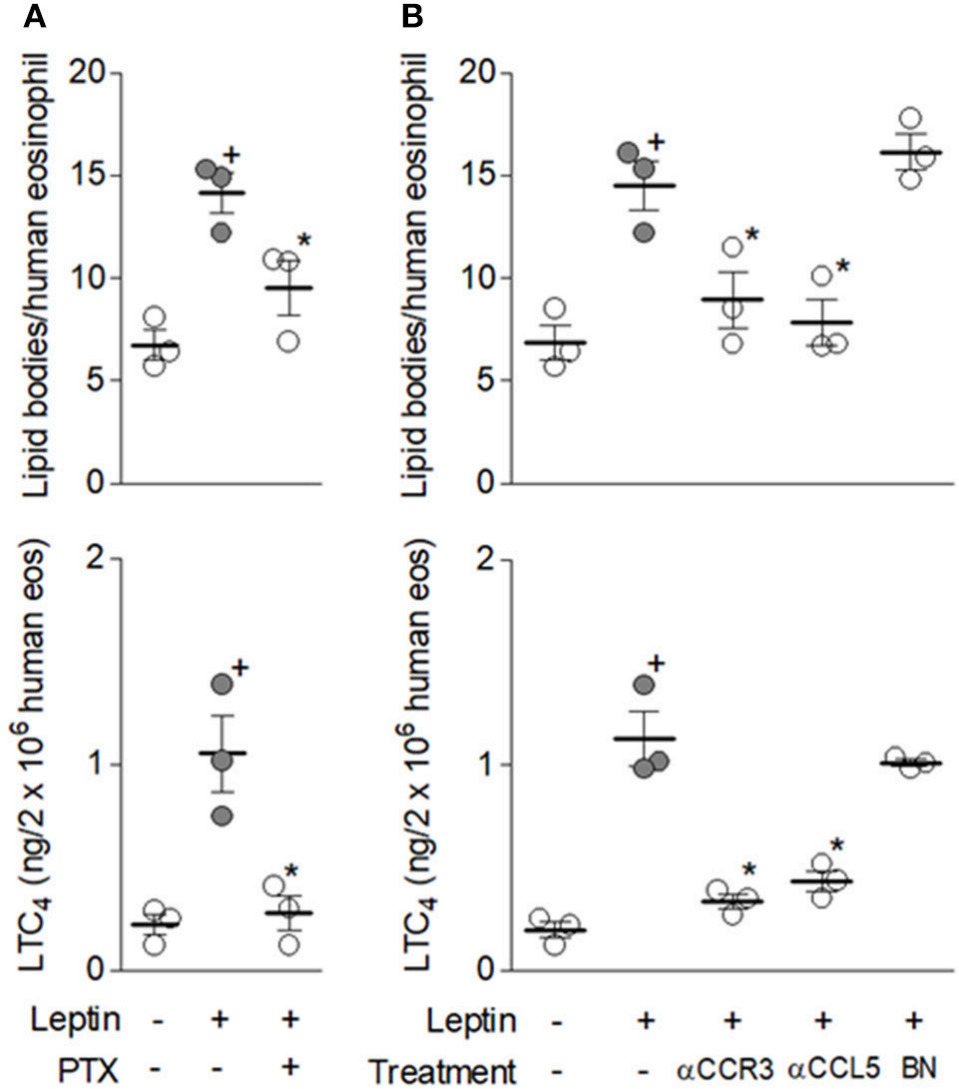
G protein-coupled CCR3 receptor activation by endogenous CCL5 mediates leptin-induced lipid body-driven LTC_4_ synthesis. In **(A)** human eosinophils were pretreated with pertussis toxin (PTX; 100 ng/mL). In **(B)** human eosinophils were pretreated with neutralizing anti-CCR3 or anti-CCL5 antibodies (both at 10 μg/mL) or with the PAF receptor antagonist BN52021 (10 μM). All pretreatments were added 30 min before stimulation with hr leptin (50 nM) for 1 h. Lipid body biogenesis was evaluated in osmium-stained cells and LTC_4_ production in cell-free supernatants by EIA kits. Values are expressed as the mean ± SEM of at least three distinct donors. + *p* < 0.05 compared with non-stimulated cells. ^*^p < 0.05 compared with leptin-stimulated eosinophils.

Pretreatment with PTX, a G protein inhibitor, was able to block both lipid body biogenesis and LTC_4_ synthesis induced by leptin within human eosinophils (Figure 4A). Whereas PI3K inhibitors effect on an ObR-mediated phenomenon was anticipated, inhibition of leptin-induced eosinophil activation by the protein pertussis (PTX) would be an unexpected outcome, since ObR is not a G protein-coupled receptor (33). In an attempt to explain PTX effect, we investigated the potential role of autocrine loops mediated by G protein-coupled receptors in leptin-induced eosinophil activation. Indeed, induction of eosinophil functions, including lipid body-driven LTC_4_ synthesis, has been shown to depend on cross-talk between eosinophil-derived mediators in an autocrine fashion (34, 35). Initially, we studied the involvement of CCL5 and PAF–two agonists of distinct G protein-coupled receptors expressed on eosinophils that are known to induce both lipid body biogenesis and LTC_4_ synthesis (22, 28) and participate in autocrine phenomena (35, 36). As shown in Figure 4B, while pretreatment with the PAF receptor antagonist, BN52021, did not interfere with lipid body biogenesis or LTC_4_ synthesis, pretreatment with anti-CCR3 or anti-CCL5 neutralizing antibodies decreased these parameters of eosinophil activation (Figure 3B). These results not only explain PTX effect on an ObR-mediated activity, but also demonstrate the involvement of CCR3/CCL5-mediated autocrine activity in leptin-induced lipid body-driven LTC_4_ synthesis within eosinophils. Moreover, this data also demonstrated that leptin triggers release of CCL5 from human eosinophils, since only an extracellular neutralization of released CCL5 by the antibody pretreatment would inhibit leptin effects. Of note, rapid CCL5 release was firstly shown within interferon-gamma-stimulated human eosinophils and further confirmed under CD4 activation by IL-16 stimulation. These studies demonstrated that CCL5 secretion was due rapid mobilization from intracellular pre-formed stores of CCL5, which is packaged within cytoplasmic granules and vesicles within eosinophils, ready for prompt secretion (35, 37).

### Co-operative signaling through DP1 and DP2 PGD_2_ receptors is also required to lipid body-driven LTC_4_ synthesis induced by leptin in eosinophils

**Figure 5:**
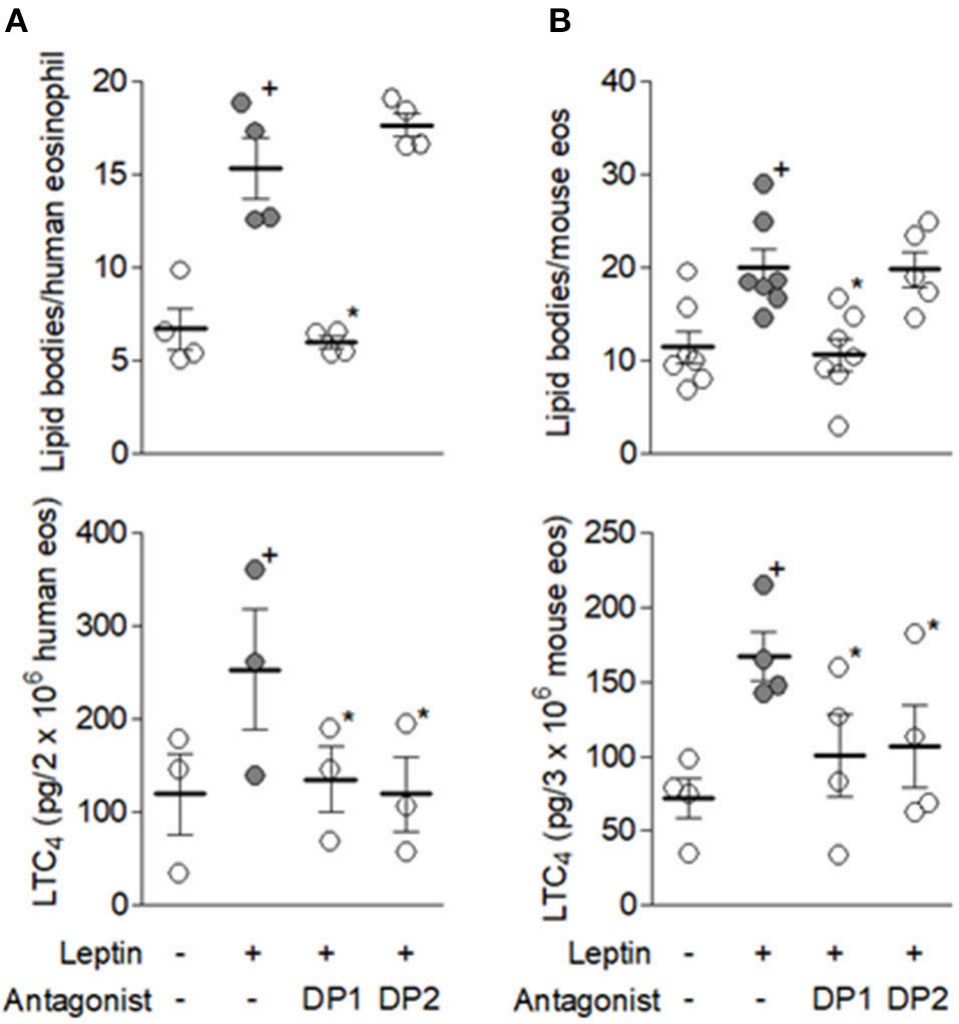
Cooperative DP1/DP2 receptor activation mediates leptin-induced lipid body-driven LTC_4_ synthesis. Human **(A)** and mouse **(B)** eosinophils were pretreated with DP1 (BWA868c; 200 nM) or DP2 (CAY10471; 200 nM) receptor antagonists for 30 min before stimulation with respectively hr leptin or mr leptin (50 nM) for 1 h. Lipid body count was evaluated in osmium-stained cells and LTC_4_ production in cell-free supernatants by EIA kits. Values are expressed as the mean ± SEM of at least three distinct donors or three different mouse bone marrow cultures. + *p* < 0.05 compared with control. ^*^p < 0.05 compared with leptin-stimulated eosinophils.

**Figure 6:**
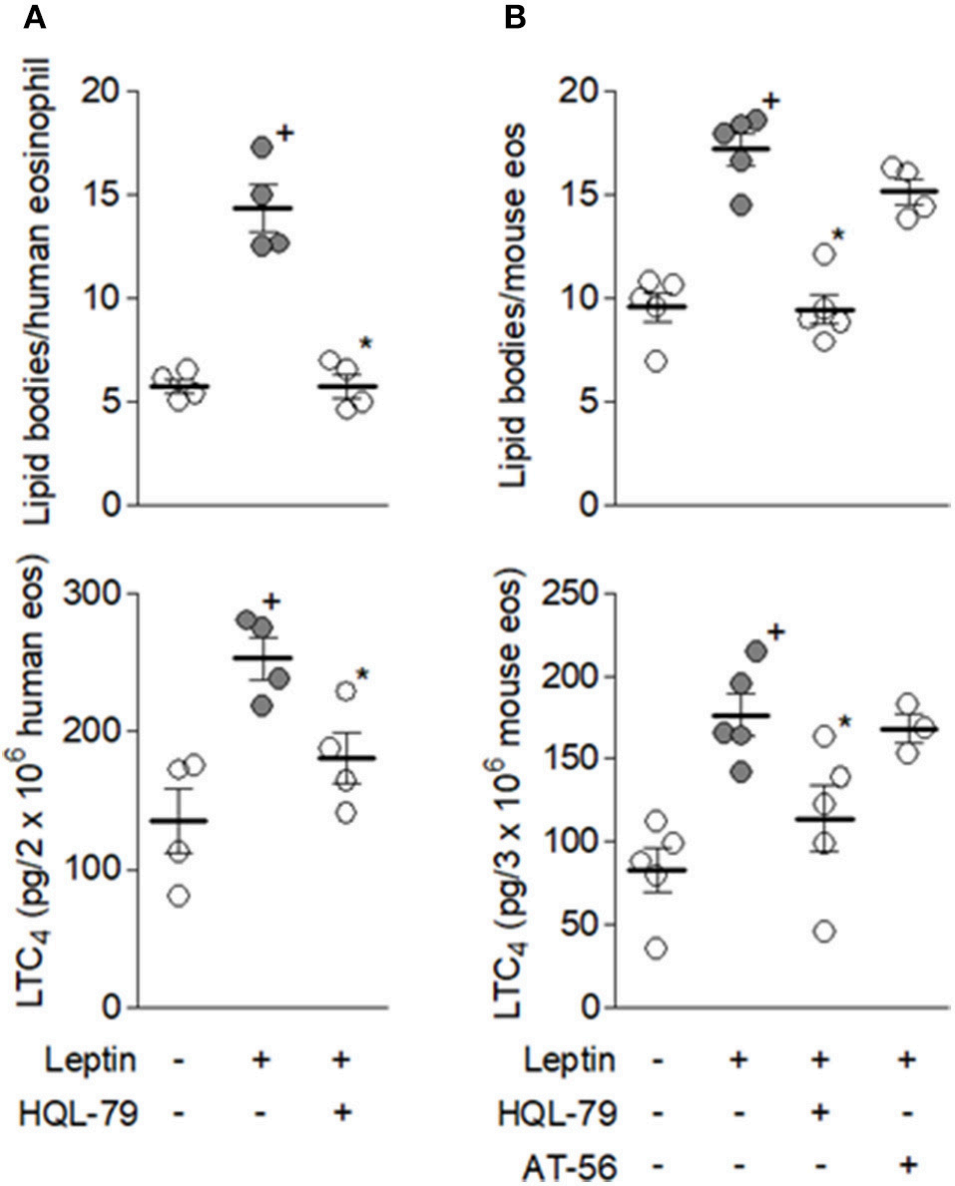
Endogenous eosinophil-derived PGD_2_ mediates leptin-induced lipid body-driven LTC_4_ synthesis. In **(A)** human eosinophils were pretreated with PGD_2_ synthesis inhibitor HQL-79 (10 μM) for 30 min before stimulation with hr leptin (50 nM) for 1 h. In **(B)** mouse eosinophils were pretreated with PGD_2_ synthesis inhibitors HQL-79 (10 μM) or AT-56 (10 μM) for 30 min before stimulation with mr leptin (50 nM) for 1 h. Lipid body count was evaluated in osmium-stained cells and LTC_4_ production in cell-free supernatants by EIA kits. Values are expressed as the mean ± SEM of at least three distinct donors or three different mouse bone marrow cultures. + *p* < 0.05 compared with control. ^*^ *p* < 0.05 compared with leptin-stimulated eosinophils.

Autocrine PGD_2_ participation on leptin-induced lipid-body driven LTC_4_ synthesis was also investigated, considering: (i) the rapid PGD_2_ synthesis and secretion triggered by leptin stimulation of human or mouse eosinophils (Figure 1); (ii) that PGD_2_ is a potent inducer of both lipid body biogenesis and LTC_4_ synthesis within human or mouse eosinophils (38, 39); (iii) PGD_2_-induced effects comprise PTX-sensitive activation of G protein-coupled receptor on eosinophils (39); and (iv) eosinophil-derived PGD_2_ have previously shown autocrine activity on CCR3-activated eosinophils (29). Identical to the inhibitory pattern observed on eosinophils stimulated by PGD_2_ itself (39), lipid body biogenesis induced by leptin was inhibited only by DP1 receptor antagonist BWA868c, while LTC_4_ synthesis was blocked by both BWA868c and DP2 receptor antagonist Cay10741 within leptin-stimulated human (Figure 5A) or mouse eosinophils (Figure 5B), indicating the same cooperation between DP1 and DP2 receptors responsible for eosinophil activation triggered by exogenous PGD_2_ appears to take place under leptin stimulation. To ascertain that an endogenously-produced PGD_2_ was mediating leptin-induced lipid body-driven LTC_4_ synthesis, eosinophils were pretreated with the specific inhibitor of hematopoietic prostaglandin D (PGD) synthase, HQL-79, which was able to inhibit both lipid body biogenesis and LTC_4_ production induced by leptin stimulation of human (Figure 6A) or mouse eosinophils (Figure 6B). Of note, an inhibitor of lipocalin-type prostaglandin D synthase (L-PGDS), AT-56, had no impact on both lipid bodies biogenesis and LTC_4_ production (Figure 6B). Altogether the data prove that simultaneous activation of PGD_2_ G-coupled receptors DP1 and DP2 by an eosinophil-derived PGD_2_ corresponds to a second autocrine loop mediating leptin-elicited lipid body-driven LTC_4_ synthesis by eosinophils.

### Activation of CCR3 by endogenous CCL5 precedes and triggers autocrine activity of PGD_2_ that culminates in leptin-elicited LTC_4_ synthesis

**Figure 7:**
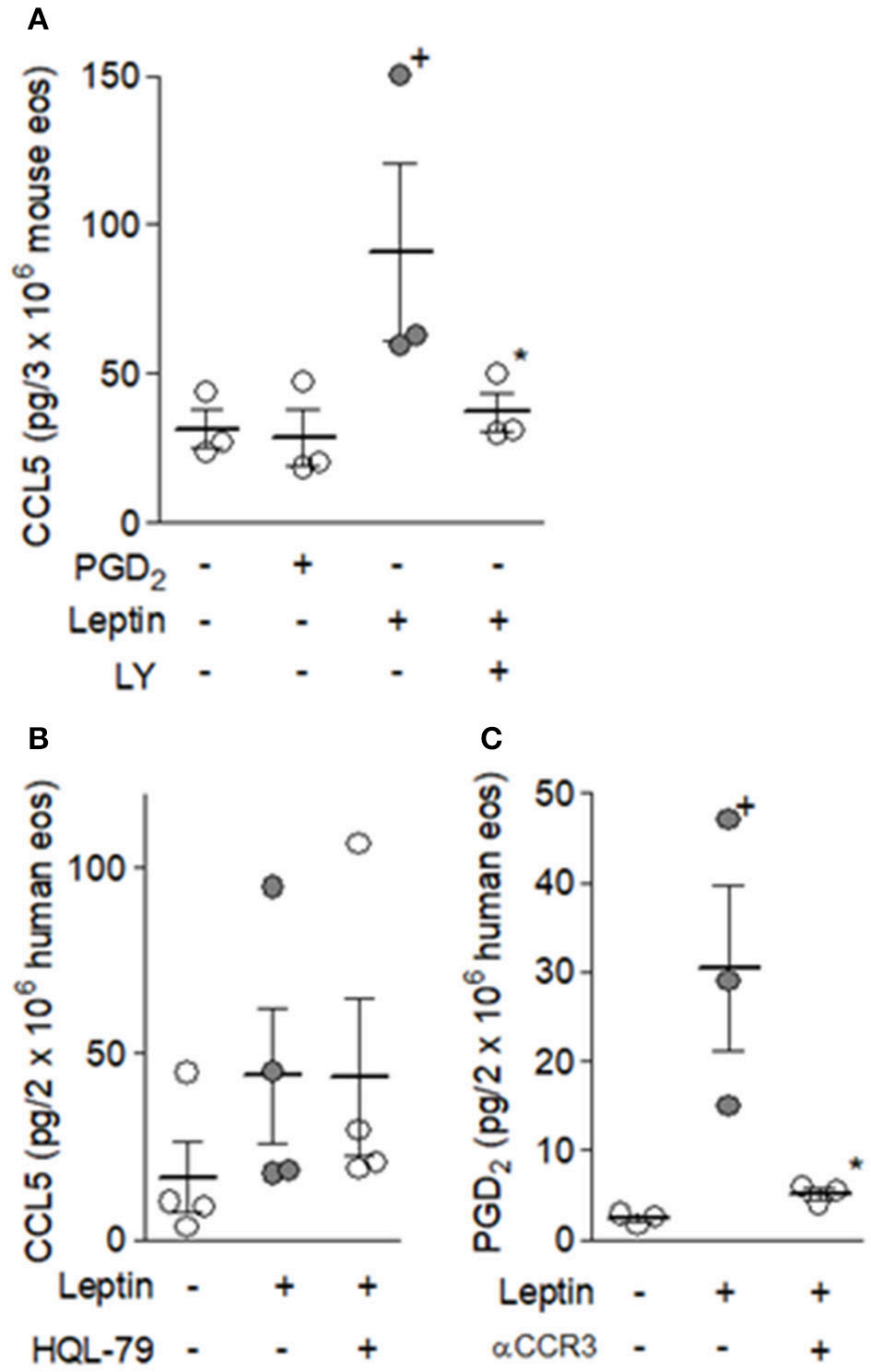
Autocrine CCR3 activation by eosinophil-derived CCL5 elicits PGD_2_ synthesis in response to leptin stimulation of eosinophils. In **(A)** mouse eosinophils were stimulated for 1 h with PGD_2_ (25 nM) or mr leptin (50 nM); mr leptin-stimulated cells were pretreated LY294002 (10 μM) for 30 min. In **(B)** and **(C)** human eosinophils were pretreated with HQL-79 (10 μM) or neutralizing anti-CCR3 antibody (10 μg/mL) for 30 min before stimulation with respectively hr leptin (50 m nM) for 1 h. CCL5 secretion was evaluated in cell free supernatants by ELISAand PGD_2_ production in cell-free supernatants by EIA kits. Values are expressed as the mean ± SEM of at least three distinct donors or three different mouse bone marrow cultures. + *p* < 0.05 compared with control. ^*^p < 0.05 compared with leptin-stimulated eosinophils.

**Figure 8:**
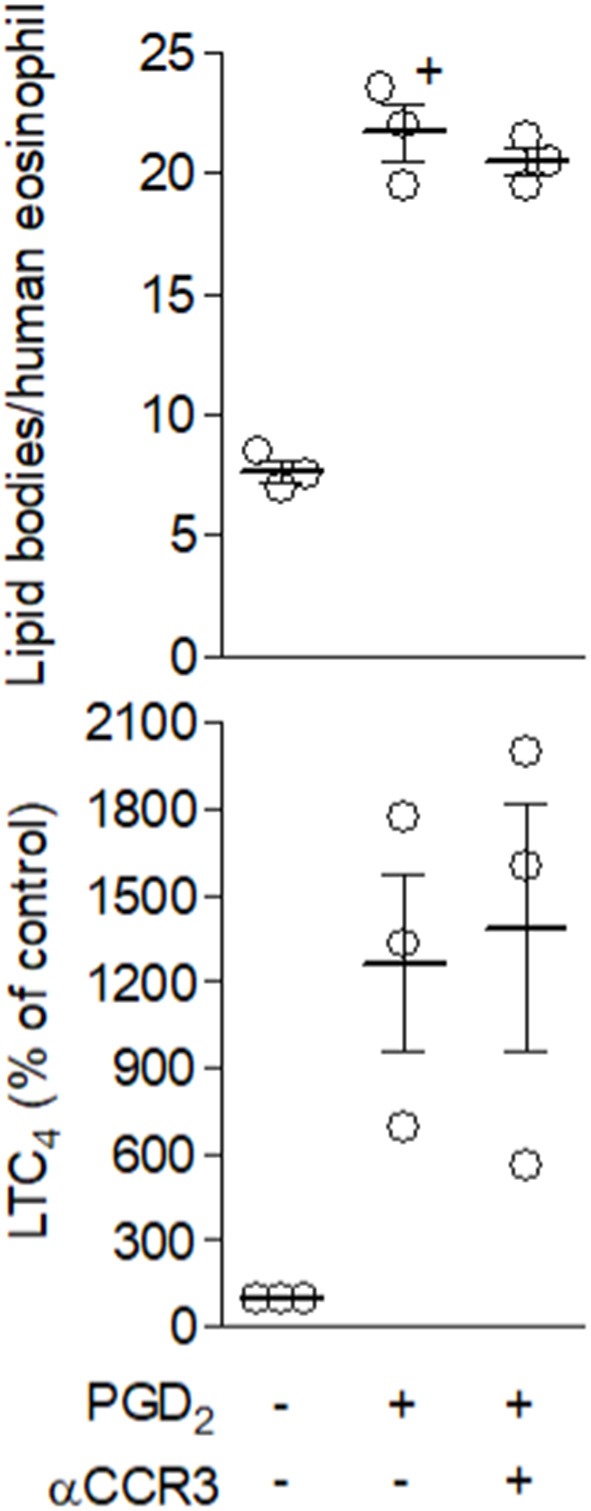
Autocrine CCR3 activation does not play a role in eosinophil activation by PGD2. Human eosinophils were pretreated with neutralizing anti-CCR3 antibody (10 μg/mL) for 30 min before stimulation with PGD_2_ (25 nM) for 1 h. Lipid body count was analyzed in osmium-stained cells and LTC_4_ production in cell-free supernatants by EIA kits. Values of top panel are expressed as the mean ± SEM of at least three distinct donors. + *p* < 0.05 compared with control. In bottom panel, normalized values show mean ± SEM and individual % percentage of LTC_4_ control levels, which were for each of the three donors analyzed 10, 75, and 656 pg/2 × 10^6^ human eosinophils.

Inasmuch as we have established that leptin stimulation triggers signaling pathways comprising at least two extracellular stimulatory events of G protein coupled-receptors by molecules secreted by the eosinophils themselves, specifically CCL5- and PGD_2_-activating CCR3 and DP1/DP2 receptors, the sequence of these events was then studied. The data shown in Figures 7, 8 collectively identify CCL5/CCR3 step as an initial cellular event that determines subsequent PGD_2_/DP receptors-mediated step of the leptin-induced lipid body-driven LTC_4_ synthesis. First, we found that leptin, but not PGD_2_, appears to be able to induce PI3K activation-dependent secretion of CCL5 to the extracellular space of mouse eosinophils (Figure 7A). Figure 7B shows similar phenomenon for leptin-stimulated human eosinophils. Even though leptin-induced CCL5 release by human eosinophils was not found statistically significant, a clear tendency of CCL5 secretion triggered by leptin can be observed (Figure 7B). Indeed, CCL5 secretion by leptin-stimulated human eosinophils had been shown here in an indirect manner by the effectiveness of anti-CCL5 antibody treatment in inhibiting leptin-induced human eosinophil activation (Figure 4B); addition of neutralizing antibody molecules to viable and bioactive cells, such as leptin-stimulated eosinophils, can only target secreted/extracellular CCL5, inasmuch as intact cells are impermeable to antibody molecules. Second, pretreatment with HQL-79 failed to inhibit leptin ability to induce CCL5 release from human eosinophils (Figure 7B), while pretreatment with anti-CCR3 antibody inhibits leptin ability to induce PGD_2_ production (Figure 7C). Finally, the treatment with anti-CCR3 antibody failed to inhibit PGD_2_-induced lipid body formation and LTC_4_ production (Figure 8), indicating that the PGD_2_-induced eosinophil activation, a phenomenon known to depend on cooperation between both DP1 and DP2 expressed on eosinophils (39), is not mediated by CCR3-driven autocrine loop.

## Discussion

Eosinophils have emerged recently as key housekeeping cells of adipose tissue physiology (2, 11). The mechanisms of homeostatic eosinophils on healthy adipose tissue depend on eosinophils' abilities to secrete mediators that control resident adipose tissue cells, including IL-4/macrophages (7–9) and cathecolamines/adipocytes (13) paracrine circuits. However, the physiologically relevant adipose tissue-derived stimuli for proper eosinophil activation remain elusive. Leptin emerges as preferential candidate in view of the understanding that (i) eosinophils are resident cells within adipose tissue (7), (ii) leptin is continuously produced by adipocytes, and (iii) eosinophils express biologically active leptin receptors (15). Thus, studies characterizing the role of leptin in the activation and effector function of eosinophils may unveil new pathways and molecules involved in the eosinophil-driven immune-regulation of adipose tissue.

**Figure 9:**
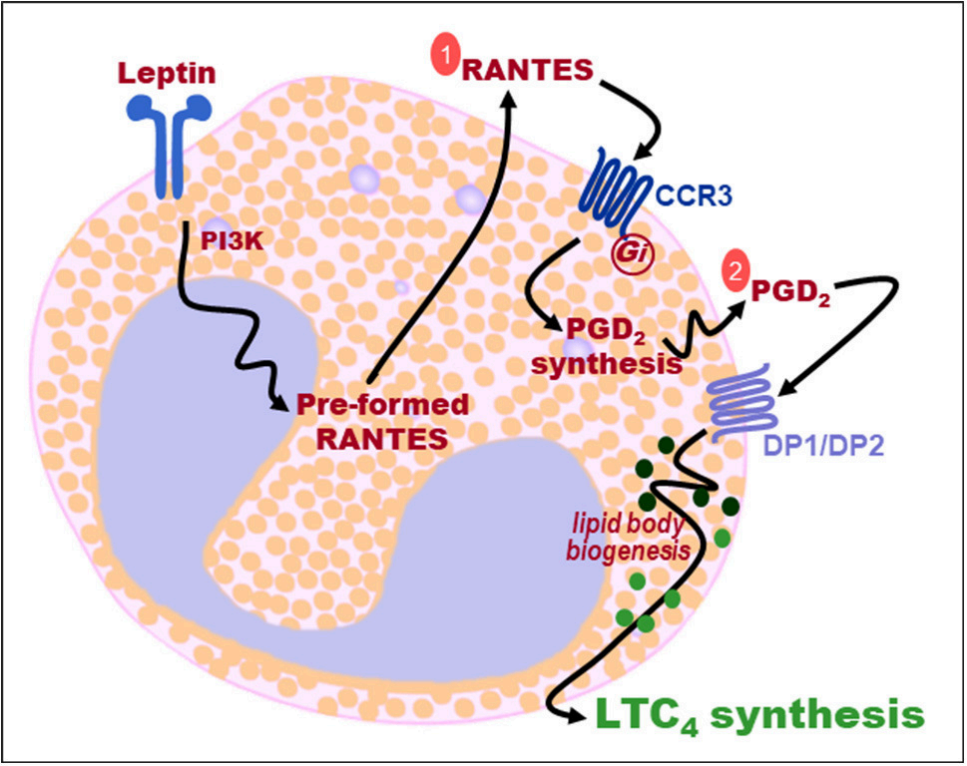
Sequential two-step autocrine mechanism of leptin-induced lipid body-driven LTC_4_ synthesis within eosinophils. Leptin stimulation of eosinophils activates PI3K signaling, rapid RANTES secretion, autocrine activation of CCR3 receptors by RANTES (step 1), PGD_2_ synthesis, autocrine activation by PGD_2_ of both DP1 and DP2 receptors (step 2), that cooperate to trigger lipid body biogenesis and lipid body-compartmentalized LTC_4_ synthesis.

Here we demonstrated that leptin has distinctive regulatory roles in activating arachidonic acid metabolism within eosinophils. Leptin activates eosinophils through a multi-step pathway that sequentially involves secretion and autocrine signaling of RANTES and PGD_2_, through CCR3 and DP1/DP2 activation respectively, for LTC_4_ production (Figure 9). It is noteworthy that the whole picture of eosinophil secretory capability upon stimulation with leptin or other adipose tissue-derived molecules is far from fully characterized. Therefore, the primary relevance of our study is the identification of eicosanoids as leptin-induced eosinophil-derived mediators.

Our study is the first to unveil the ability of leptin to induce synthesis of a prostanoid. PGD_2_ secreted by eosinophils upon leptin stimulation proved to be a bioactive molecule displaying autocrine activity able to trigger rapid LTC_4_ synthesis. The sensitivity to HQL-79 (but not to ATL-56) treatment showed the involvement of H-PGDS, rather than L-PGDS, in leptin-induced synthesis of PGD_2_ by eosinophils. Of note, expression of H-PGDS by adipose macrophages was shown to positively correlate with healthy adipose tissue features (40), while PGD_2_ synthesis dependent on adipocyte-expressed L-PGDS are related to inflammatory pro-adipogenic functions (41–44). Similar to leptin, CCR3 direct activation by exogenous chemokines, including eotaxin or RANTES, also induces HQL-79-sensitive H-PGDS-driven PGD_2_-synthesizing activity, a rapid (within few minutes) phenomenon which is followed by PGD_2_-driven autocrine induction of LTC_4_ synthesis by eosinophils (29). This piece of information delivers the cellular event lacking to complete the sequence of leptin-elicited signaling steps of LTC_4_ synthesis within eosinophils: (i) leptin receptor stimulation, (ii) PI3K activation, (iii) rapid secretion of pre-formed RANTES, (iv) autocrine CCR3 activation by extracellular RANTES, (v) rapid H-PGDS-driven synthesis of PGD_2_, (vi) autocrine activation of PGD_2_ receptors DP1/DP2, and finally (vii) lipid body-compartmentalized LTC_4_ synthesis (Figure 8). Therefore, while studying LTC_4_ synthesis, we found out that leptin stimulation triggers secretion of at least two more active molecules, RANTES and PGD_2_, which may have additional functions besides triggering LTC_4_ synthesis described here.

Within adipose tissue, leptin produced by mature adipocytes continuously may induce synthesis of bioactive PGD_2_ by eosinophils. Secreted PGD_2_ may act to activate eosinophils to release LTC_4_ in autocrine fashion, and may also stimulate nearby adipose tissue cells in a paracrine fashion. Acting on DP receptors expressed by resident cells in adipose tissue, leptin-induced PGD_2_ can down-regulate production of leptin (45), trigger secretion of Th2 cytokines IL-5 as well as IL-4 by ILC2s (46, 47), or polarize macrophages toward a M2 anti-inflammatory state in an autocrine fashion (40)—all adipose housekeeping mechanisms of metabolic syndrome evasion.

In peritoneal macrophages, stimulation of leptin receptors and subsequent PI3K activation regulates arachidonic acid metabolism by 5-LO to synthesize LTB_4_–a pro-inflammatory mediator known for its potent neutrophilotactic activity (48). Distinctly, 5-LO couples with LTC_4_ synthase to generate LTC_4_ within properly stimulated eosinophils, a highly regulated intracellular event that is known to be compartmentalized within eosinophil cytoplasmic lipid bodies. Concurring, enhanced LTC_4_ synthesis by circulating granulocytes (including eosinophils) positively correlates with increased leptin levels (49). Here, we showed that leptin stimulation of eosinophils is capable of rapid assembly of the enzymatic LTC_4_-synthesizing machinery within newly formed lipid bodies that culminates with detection of extracellular LTC_4_ within 1 h of stimulation. PI3K activation by leptin, follows through two sequential steps of autocrine activity, involving CCR3 and then DP1/DP2 activation, already described as capable individually to trigger lipid body-driven LTC_4_ synthesis within eosinophils (26, 29, 39). It is noteworthy that PI3K activation also mediates CCR3-driven LTC_4_ synthesis (26), reinforcing the role of this pathway to leptin-induced lipid body-driven arachidonic acid metabolism.

LTC_4_ and its extracellular metabolites LTD_4_ and LTE_4_ are classically recognized by their major pro-inflammatory effector roles in allergic conditions and asthma (50). There are very few reports addressing LTC_4_ roles on adipose tissue regulation, but they indicate potential homeostatic functions. It has been shown that LTC_4_ is able to: (i) potentiate ILC2 activation with increased release of IL-5 (51), which can control homeostatic eosinophilia of adipose tissue (12); and (ii) induce IL-4 secretion from eosinophils (52, 53), therefore with indirect stimulatory impact in M2 macrophage phenotype.

Our findings point to the need for detailed studies considering the adipose tissue environment as a source of molecules that trigger fine-tuned eosinophil activation, that instead of inducing release of eosinophil potentially pro-inflammatory, or even toxic, granule contents (2, 54), do elicit secretion of eosinophil-derived immunomodulatory molecules with homeostatic impact on adipose tissue, like IL-4, cathecolamines and possibly eicosanoids, H-PGDS-driven PGD_2_ and LTC_4_. Leptin appears to be one of these very special eosinophil activators that may also include PGD_2_ and LTC_4_ themselves. Indeed, even intracellular LTC_4_ can function as an intracrine signal that regulates IL-4 secretion from eosinophils (53, 55), therefore placing the leptin/eosinophil/PGD_2_/LTC_4_ axis as a potential determining factor in immune-mediated homeostasis of adipose tissue.

## Author contributions

All authors had critically revised and approved the final version of the manuscript. NA, TL-G, CM-M, and CB-M performed the conception, designed and performed the experiments, analyzed and interpreted data, and wrote the manuscript draft. MG-A and GS-A participated in the data acquisition, analysis, and interpretation of the data. CC, BD, PW and PB participated in the conception, design, analysis, and interpretation of the work.

### Conflict of interest statement

The authors declare that the research was conducted in the absence of any commercial or financial relationships that could be construed as a potential conflict of interest. The handling editor declared a shared affiliation, though no other collaboration, with several of the authors NA, TL-G, MG-A, CC, BD, CB-M.

## Abbreviations

5-LO: 5-lipoxygenase
COX: cyclooxygenase
EIA: enzyme immuno assay
LTC4: leukotriene C4
PGD2: prostaglandin D2
RANTES: Regulated on Activation Normal T Cell Expressed and Secreted.
